# Involvement of Neurons in the Nonhuman Primate Anterior Striatum in Proactive Inhibition

**DOI:** 10.1523/JNEUROSCI.0866-24.2024

**Published:** 2024-11-04

**Authors:** Atsushi Yoshida, Okihide Hikosaka

**Affiliations:** Laboratory of Sensorimotor Research, National Eye Institute, National Institutes of Health, Bethesda, Maryland 20892

**Keywords:** basal ganglia, macaque, proactive inhibition, saccade, striatum

## Abstract

Behaving as desired requires selecting the appropriate behavior and inhibiting the selection of inappropriate behavior. This inhibitory function involves multiple processes, such as reactive and proactive inhibition, instead of a single process. In this study, two male macaque monkeys were required to perform a task in which they had to sequentially select (accept) or refuse (reject) a choice. Neural activity was recorded from the anterior striatum, which is considered to be involved in behavioral inhibition, focusing on the distinction between proactive and reactive inhibitions. We identified neurons with significant activity changes during the rejection of bad objects. Cluster analysis revealed three distinct groups, of which only one showed increased activity during object rejection, suggesting its involvement in proactive inhibition. This activity pattern was consistent irrespective of the rejection method, indicating a role beyond saccadic suppression. Furthermore, minimal activity changes during the fixation task indicated that these neurons were not primarily involved in reactive inhibition. In conclusion, these findings suggest that the anterior striatum plays a crucial role in cognitive control and orchestrates goal-directed behavior through proactive inhibition, which may be critical in understanding the mechanisms of behavioral inhibition dysfunction that occur in patients with basal ganglia disease.

## Significance Statement

This study revealed a group of neurons in the anterior striatum that plays a crucial role in cognitive control by actively participating in the rejection of unfavorable choices. Contrary to previous belief, these neurons were involved in proactive inhibition (i.e., the process of discarding unnecessary options), instead of suppressing automatic responses, to achieve a goal. This distinction is vital for understanding the mechanisms by which the brain makes decisions and may have implications for addressing neurological disorders associated with impaired decision-making and inhibitory control. Our findings provide new insights into the neural mechanisms underlying goal-directed behavior and highlight the importance of the anterior striatum in orchestrating complex cognitive functions.

## Introduction

Achieving goals requires selecting the desired action and suppressing other compelling actions. The inhibition of actions is multifaceted, and there are distinctions between automatic and volitional suppressions, as well as reactive and proactive suppressions (Sumner et al., 2007; Boy et al., 2010; Zandbelt and Vink, 2010; Aron, 2011; Verbruggen and Logan, 2008; Jahanshahi et al., 2015). Reactive inhibition is often triggered by external stimuli and is habitual and reflexive. For instance, a red traffic light prompts us to stop walking or apply the brakes while driving. In contrast, proactive inhibition is volitional and goal-directed, such as when students resist the temptation to play to focus on their studies. Understanding the specific brain regions that control these inhibitory functions is crucial to advancing our knowledge of cognitive control mechanisms.

Previous functional brain imaging studies in humans and physiological studies in experimental animals, including rodents and nonhuman primates, have implicated an indirect pathway in the basal ganglia for these inhibitory functions. This pathway includes the striatum (caudate nucleus and putamen), globus pallidus external segment (GPe), subthalamic nucleus (STN), and globus pallidus internal segment or substantia nigra pars reticulata (SNr). Specifically, the striatum and the STN have been associated with reactive inhibition (Aron and Poldrack, 2006; Aron et al., 2007; Li et al., 2008), and the striatum has been suggested to play a crucial role in proactive suppression (Vink et al., 2005; Chikazoe et al., 2009; Jahfari et al., 2012; Majid et al., 2013; Smittenaar et al., 2013; Schel et al., 2014). Neurophysiological studies involving nonhuman primates initially demonstrated the involvement of the striatum in reactive inhibition (Apicella et al., 1992; Hori et al., 2009), while subsequent studies have indicated the involvement of both the striatum and GPe in proactive inhibition (Yoshida and Tanaka, 2009, 2016; Watanabe and Munoz, 2010). However, the antisaccade task used in these studies, which requires both the facilitation of desired choices and the inhibition of unwanted behaviors, makes it challenging to discern specific neural representations. To address this issue, we developed a choice task with sequential presentation of alternatives, allowing for the separation of choice and rejection actions. By recording neuronal activity from the GPe from macaque monkeys while performing this task, we found a subset of GPe neurons significantly changed their neuronal activity when rejecting an object choice (Yoshida and Hikosaka, 2023). Furthermore, by combining it with a fixation task that required inhibition of the reflexive saccades (reactive inhibition), we showed that this group of neurons in the GPe may be involved in proactive inhibition.

Our previous study (Yoshida and Hikosaka, 2023) found most neurons involved in proactive inhibition in the dorsal part of the GPe; thus, we recorded neural activity mainly in the anterior and body parts of the striatum, which were thought to project to this area. The purpose of this study was to investigate whether the neurons in the striatum were involved in proactive inhibition using the choice and fixation tasks used in our previous study. Given that the firing rates of the GPe neurons decreased during proactive inhibition, we focused our neural activity recordings on the medium spiny neurons in the striatum that sent inhibitory projections to the GPe (Precht and Yoshida, 1971).

## Materials and Methods

### Animal preparation

All animal care and experimental procedures were approved by the National Eye Institute Animal Care and Use Committee and complied with the Public Health Service Policy on Human Care and Use of Laboratory Animals.

Two male macaque monkeys (*Macaca mulatta*, aged 9 years, 9–10 kg body weight), referred to as Monkeys C and S, were used. After the monkeys were trained to sit in the chair voluntarily, they underwent surgery under general anesthesia using standard sterile techniques for the implantation of a plastic head holder and recording chambers. They were sedated with ketamine hydrochloride and dexmedetomidine, and atropine sulfate was administered intramuscularly. The monkeys were intubated and maintained with isoflurane using an anesthetic ventilator. During the surgery, oxygen saturation, end-tidal carbon dioxide, noninvasive blood pressure, electrocardiogram, and body temperature were continuously monitored. The head holder and recording chambers were securely affixed to the craniums of both subjects with ceramic screws and dental acrylic. Pain relief was provided with analgesics during the surgical procedure and for 5 d afterward. Training for oculomotor tasks commenced after surgical recovery. The daily water intake of the monkeys was controlled to motivate them to perform behavioral tasks. The monkey heads were immobilized using a primate chair throughout the training and experiments. An eye-tracking device (EyeLink 1000; SR Research) recorded the eye movements at a frequency of 1,000 Hz.

### Experimental design

The trials were conducted in a secluded, light-controlled, and soundproof environment. The participants were positioned on a restraint device facing an interactive display. Visual stimuli were projected onto the screen in front of the participants using an active matrix liquid crystal display projector (PJ658; ViewSonic). A custom-made C++-based experimental data acquisition system (Blip; available at http://www.robili.sblip/) was used to control the behavioral tasks. This study employed two distinct behavioral tasks: choice and fixation.

### Choice and fixation tasks

**Figure 1.**
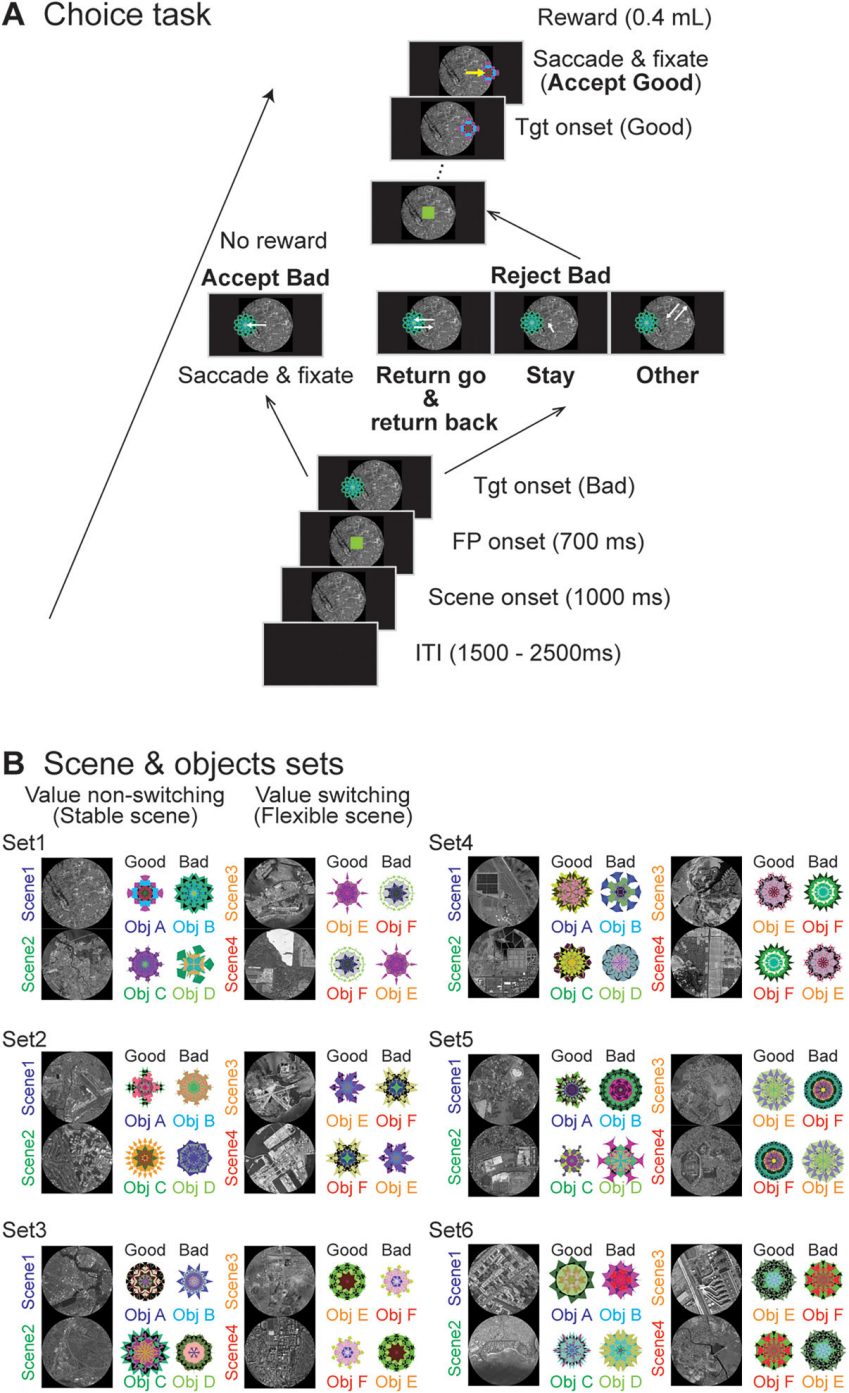
Task procedures. ***A***, Procedure for the choice task. The monkeys are considered to have chosen a fractal object presented to them if they make a saccade toward it and fixated on it for >400 ms (Accept). They reject the object (Reject) by making a return saccade (Return), looking around the center point (Stay), or making a saccade away from the bad object (Other). ***B***, All six sets used for recording from a single neuron. For neuronal recording from an individual neuron, only one set is used. One set includes four scenes, and each scene has one good (rewarded) and one bad (nonrewarded) fractal object during the choice task. In Scenes 1 and 2, the object values are always the same (stable), while the same two objects are used in Scenes 3 and 4, but their values are replaced (flexible) in each scene.

We used the choice and fixation tasks. In the choice task, circular grayscale aerial images (radius, 25°) from OpenAerialMap, referred to as scenes, were presented as the background on the screen (Fig. 1*A*). Computer-generated multicolored fractal objects were utilized as targets (radius, 5°; Yamamoto et al., 2013). One of the six sets of scenes was randomly chosen during each single-unit recording session (Fig. 1*B*). Each set contained four different scenes, and each scene included two objects: one “good” fractal object (associated with reward delivery) and one “bad” object (not associated with a reward). The value rationings to the stimuli remained unchanged in the “stable” scenes (1 and 2) but were changeable in the “flexible” scenes (3 and 4). This design aimed to discern the relationship of these neuronal responses to the features of the objects or their value assignments.

The task sequence started with the unveiling of a scene following a preparatory period (1,500–2,000 ms). The subject saw the scene freely before a fixation point (FP) emerged at the center of the display (1,000 ms). After maintaining the stare at the FP (700 ms), one of the fractal objects, either good or bad, randomly and sequentially appeared as a target after the FP offset. The target was randomly shown in one of six positions (eccentricity, 15°; angles, 0, 45, 135, 180, 225, and 315°). If the participant made a saccade toward the target and fixated on it for >400 ms, this action was recorded as object acceptance (accept), resulting in the delivery of a reward (0.4 ml) for a good object. No reward was given for bad objects. If the subject kept looking around the center point (stay), made a saccade to the target but returned their gaze to the center point within 400 ms (return), or looked away from the target (other), the FP would reappear. These actions were considered when the participants refused to select an object (rejected).

The range of the target was set to a square (8° per side). If the eye position of the subject went outside the target range for <400 ms after making a saccade to the target, the target was gone and an FP appeared again. As a result, when the monkeys rejected bad objects by staying or other means, the waiting time for the next FP to be presented was 1,000 ms, whereas the waiting time was <1,000 ms if they rejected bad objects by returning. When the subject rejected bad objects by returning, two saccades occurred: one was toward the object (return go), and the other was from the object to the center (return back). Another target was presented when the subjects were gazing at the FP. This procedure was repeated 15 times until the participants selected the target object.

To train the monkeys to learn the rules of the choice task, we first used a different set from the six sets employed in the current study. As in Scenes 1 and 2, we used a block condition in which the values of good and bad objects remained constant (stable condition). At the beginning of training, the monkeys did not know which object was rewarded, so they accepted (made a saccade and fixated on) both good and bad objects. After some time, they began to make a saccade toward the bad object but did not fixate on it, instead immediately returning to the center point (return), or they did not saccade and continued to fixate around the center point, waiting for the next object to be presented (stay).

Once the percentage of correct responses in the block condition using only Scenes 1 or 2 exceeded 90%, we trained the monkeys in the flexible condition (Scenes 3 and 4), where the values of objects switched depending on the scene. Initially, we trained the monkeys using only the Scene 3 condition, where Object E was a good object and Object F was a bad object. The monkeys quickly learned to accept Object E and reject Object F. When their success rate exceeded 90%, they were then trained using only the Scene 4 condition, in which the object values were reversed. Since the monkeys had learned that Object E was a good object and Object F was a bad object, they initially accepted Object E and rejected Object F in the Scene 4 condition. However, as they did not receive juice as a reward, they gradually began to reject Object E and accept Object F. When the monkeys achieved >90% success in the Scene 4 condition, we reintroduced training using only the Scene 3 condition. The monkeys gradually understood the significance of the presented scenes and were able to switch object selection immediately after the scene condition changed.

After completing the above training, we trained the monkeys under a condition in which Scenes 1–4 were presented randomly. Following these training sessions, both monkeys achieved success rates exceeding 90% within a few days, even when using the other sets (four scenes and six objects).

The aim of the fixation task was to elucidate whether the neuronal activity was related to suppressing saccades to presented objects. This task followed the choice task in which individual neurons were isolated. In this task, both fractal objects (one good and one bad object) from Scene 1 of the choice task were displayed alternately 2–4 times to the left or right of the FP; each was displayed for 400 ms with a 400 ms interstimulus interval. The subjects were required to keep their gaze on a white fixation point (FP) during the simultaneous display of the fractal object. A reward (0.4 ml) was given 300 ms after the presentation of the last object. Saccades toward the object stimuli were considered fixation break errors.

### MRI

After the surgery to implant recording chambers, magnetic resonance imaging (MRI) was performed to delineate cerebral structures and apertures in the grid with a gadolinium-enhanced contrast medium (Magnevist, Bayer Healthcare Pharmaceuticals) introduced into the chambers. The recording sites were pinpointed and reconstructed from the MRI data using a high-definition 3T MR apparatus (MAGNETOM Prisma; Siemens Healthcare). This process involved the acquisition of both three-dimensional T1-weighted (T1w, MPRAGE) and T2-weighted (T2w, SPACE) sequences, each with a uniform voxel dimension (0.5 mm; Mugler et al., 2000).

To illustrate the locations of the recorded task-related neurons in the striatum, we segmented the region of the striatum automatically using a pipeline with the AFNI software (Cox, 1996; Seidlitz et al., 2018; Jung et al., 2021). Each monkey T1w image was aligned to a standard NIMH macaque templates (NMT v2.0) using the AFNI macaque pipeline. Then, the subcortical atlas of the rhesus macaque (SARM) for NMT (Hartig et al., 2021) was inversely transformed into a native T1w image. We compared the segmented images of the striatum with the quantitative susceptibility mapping images of the subjects to confirm the validity of the former; the former can visualize the structure of the striatum more clearly than T1w and T2w images (Yoshida et al., 2021; Dadarwal et al., 2022). We used the 3D Slicer software (version 5.2.2; Fedorov et al., 2012) and Blender software (version 3.5) to visualize the locations of the recorded task-related neurons.

### Neuronal recording procedure

Neuronal activity was captured via single-unit recordings with epoxy- or glass-coated tungsten electrodes with impedances between 1 and 9 MΩ (Frederick Haer & Co.; Alpha Omega Engineering). The electrodes were carefully positioned within the cerebral tissue using a precision oil-driven micromanipulator (model MO-973A; Narishige) that maneuvered the electrode through a guide tube made of 23-gauge stainless steel. The neural signals were amplified, filtered (0.3–10 kHz; A-M Systems), and collected at 40 kHz. Single neurons were isolated online using custom voltage–time window discriminator software, a function of Blip. First, the threshold was set to remove signals with small amplitudes, which were considered noise. Next, multiple inclusion boxes (selecting waveforms passing through these boxes) and rejection boxes (excluding waveforms passing through these boxes) were configured to isolate neural activity based on waveform shape. Once we succeeded in the isolation of neuronal signals, the choice task was initiated. The neuronal recording was continued if neuronal activity was modulated at the target onset.

To focus on the medium spiny neurons in the striatum, only neurons with baseline firing rates of <10 spikes/s were included in this study (Aosaki et al., 1995). The average baseline activity of neurons was calculated for 1,000 ms between 1,500 and 500 ms before the scene was presented. Thus, neurons with a high firing frequency exceeding 10 spikes/s were considered fast-spiking interneurons and excluded from the present study.

### Data analysis and statistical analysis

All behavioral and neurophysiological data were preprocessed with MATLAB 2022b (MathWorks).

#### Behavior

In the choice task, saccade onsets toward good and bad objects were defined as the eye speed exceeding 40°/s within 400 ms of the target onset. During the fixation task, saccades to the presented objects were detected offline. They were considered fixation break errors.

**Figure 2.**
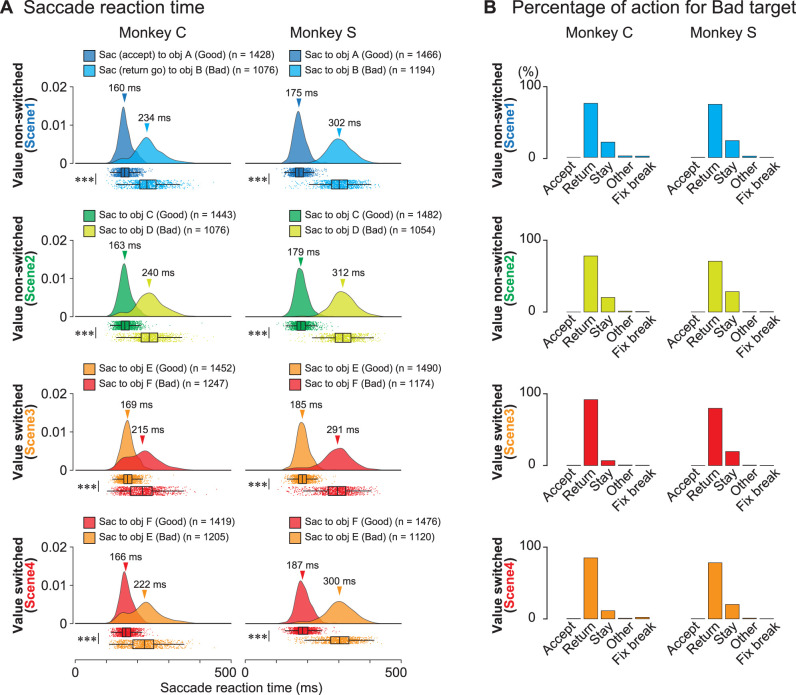
Behavioral results. ***A***, Raincloud plots of saccade reaction times for good or bad objects in Scenes 1–4 during the choice task of the two monkeys, C and S. In each panel, the top “cloud” illustrates the probability distribution of reaction times, and the bottom “rain” shows the raw plots of individual reaction times. In all the scenes, the reaction times of the two monkeys toward good objects are significantly shorter than those toward bad objects (*p* = 0 for all scenes for both monkeys, Welch's *t* tests). ***B***, Proportions of selected actions, Accept, Return, Stay, Other, or fixation break error (Fix break) for bad objects in Scenes 1–4 during the choice task. FP, fixation point; ITI, inter-trial interval; Obj, object; Tgt, target.

Welch's *t* tests were performed for each scene condition to compare the saccade reaction times for the good and bad objects (Fig. 2*A*). During this analysis, only the reaction times of the saccades toward bad objects (return go) were included, and those for returning from the bad target (return back) were not included. To determine whether the proportion of monkeys choosing to stay for a bad object was higher for Scenes 1 and 2, where the object values remained constant, Fisher's exact test was conducted (Fig. 2*B*).

#### Classification of striatal neurons

The sample size was not determined by statistical methods. Instead, the number of recorded neurons was determined based on a previous study (Watanabe and Munoz, 2010), which recorded the activities of striatal neurons of macaques while they performed an antisaccade task. All statistical analyses were performed with the preprocessed data using the R software (version 4.2.2).

**Figure 3.**
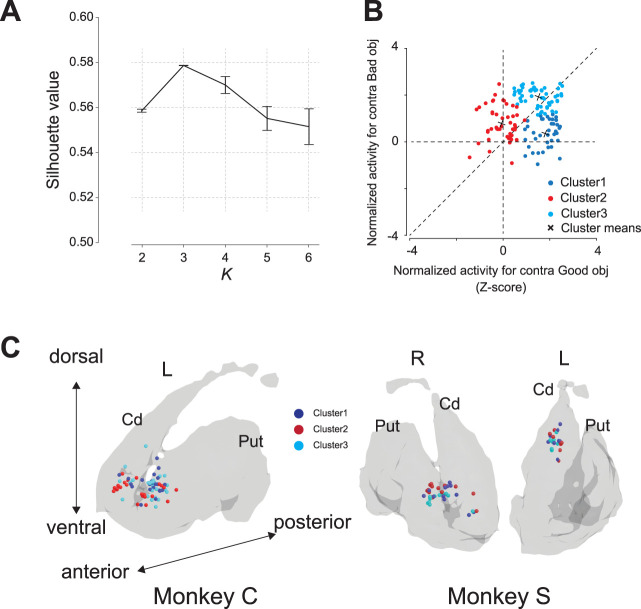
Scatterplots of clustering and recording sites in the striatum. ***A***, Result of simulation of silhouette analysis to determine the optional *K* for clustering. The *y*-axis represents the averaged silhouette number for each of the 5,000 silhouette analysis ran with *K* ranging from 2 to 6, and the error bars represent the standard deviation. ***B***, Two-dimensional scatter plots of the *Z*-transformed mean firing rate for individual neurons during 200 ms after 100 ms at the onsets of presentation of the good (*x*-axis) and bad (*y*-axis) objects. Using these two-dimensional data, the neurons are divided into three groups using clustering methods. ***C***, Reconstruction of recording site of task-related striatal neurons of the two monkeys. Cd, caudate nucleus; Put, putamen.

We employed the *k*-means clustering method to categorize neurons based on their activities when good or bad contralateral objects were presented. This process involved standardizing the activity of each neuron using a *Z*-transform. The average neuronal activity in response to the presentation of either a good or a bad object in the contralateral direction was computed. This calculation had a 200 ms window, extending from 100 to 300 ms after the onset of object presentation. To quantitatively determine the optimal number of clusters (*K*) for *k*-means classification, we simulated the silhouette values 5,000 times and examined which *K* (up to *K* = 6) produced the largest average silhouette value. We then performed a one-way analysis of variance (ANOVA) and conducted post hoc pairwise *t* tests with Bonferroni’s corrections to confirm the optimal *K* value (Fig. 3*A*,*B*). Statistical significance for the post hoc test was set at *α* = 0.05/10 with Bonferroni’s correction.

#### Comparisons of neuronal activity across conditions

The data from each neuron was aligned with the initiation of events (scene, target, and saccade onset). The time course of the neuronal response to event onset for each condition was investigated by calculating peristimulus time histograms (PSTHs) in 1 ms bins. It was smoothed with a spike density function using a Gaussian filter (*σ* = 20 ms). To visualize the neuronal activity of individual neurons on the color map, the *Z*-transformation of the activity in each neuron was performed to visualize the activities of individual neurons (Adler, et al., 2012; Kaplan et al., 2020). First, the firing rate of the baseline was calculated by averaging the firing rate within 500 ms before the scene, target, or saccade onset. Then, this baseline was subtracted from the smoothed PSTH and aligned with the initiation of events. Subsequently, a *Z*-score transformation was conducted for each PSTH; the baseline mean was subtracted from it, and the difference was divided by the standard deviation (SD).

To prevent false positives, generalized linear mixed-effects models (GLMMs) were used in this study to compare neuronal activities across conditions (Yu et al., 2022). First, we compared the full model containing explanatory variables as fixed effects for all statistical tests using the GLMM. All monkey, neuron, and experimental session IDs were included as random effects, which constituted the null model. A parametric bootstrap method was conducted to assess the goodness of fit of the models by performing 10,000 iterations and computing the *p* value based on the difference in deviance between the two models. If the full model showed a significant fit, we conducted post hoc pairwise *t* tests with Bonferroni’s corrections to further explore these differences. For GLMM, we used the *lme4* (Bates et al., 2014), *pbkrtest* (Halekoh and Højsgaard, 2014), and *emmeans* (Lenth et al., 2019) packages in RStudio.

Figure 5, *B*, *G*, and *K*, shows the comparison of neuronal activity at scene onset in the choice task. A GLMM was employed to evaluate differences in neuronal activity at scene onset. The full and null models used for the comparison are as follows:

$$\begin{array}{rl} & \mathrm{Full}\;\mathrm{Model}:\;\mathrm{Neuronal}\;\mathrm{Activity}\\ & \;∼\;\mathrm{Scene}+(1|\mathrm{monkey}\_\mathrm{ID})+(1|\mathrm{monkey}\_\mathrm{ID}:\mathrm{Neuron}\_\mathrm{ID}),\\ & \mathrm{Null}\;\mathrm{Model}:\;\mathrm{Neuronal}\;\mathrm{Activity}\\ & \;∼\;(1|\mathrm{monkey}\_\mathrm{ID})+(1|\mathrm{monkey}\_\mathrm{ID}:\mathrm{Neuron}\_\mathrm{ID}), \end{array}$$

where NeuronalActivity is the mean PSTH of individual neurons during a 200 ms period, ranging from 100 to 300 ms after scene onset; Scene (1–4) is the fixed effect; and monkey_ID and Neuron_ID are the random effects.

The level of statistical significance for the model comparison was set at *α* = 0.05.

Figure 5, *D*, *H*, and *L*, shows the comparison of neuronal activities at target onset for the four scenes during the choice task. A GLMM was conducted to examine the differences in neuronal activity at the target onset. The full and null models used for the comparison are as follows:

$$\begin{array}{rl} & \mathrm{Full}\;\mathrm{Model}:\;\mathrm{Neuronal}\;\mathrm{Activity}∼\mathrm{Scene}\times \mathrm{Value}\times \mathrm{Direction}\\ & \;+(1|\mathrm{monkey}\_\mathrm{ID})+(1|m\mathrm{onkey}\_\mathrm{ID}:\mathrm{Neuron}\_\mathrm{ID}),\\ & \mathrm{Null}\;\mathrm{Model}:\;\mathrm{Neuronal}\;\mathrm{Activity}\\ & \;∼(1|\mathrm{monkey}\_\mathrm{ID})+(1|\mathrm{monkey}\_\mathrm{ID}:\mathrm{Neuron}\_\mathrm{ID}), \end{array}$$

where NeuronalActivity is the mean PSTH of individual neurons within 200 ms from 100 ms after the target onset; Scene (1–4), Value (good vs bad), and Direction (contralateral vs ipsilateral) are the fixed effects; and monkey_ID and Neuron_ID are the random effects.

Statistical significance for the model comparison was set at *α* = 0.05. We performed six pairwise *t* tests to compare the mean neuronal activities for the good and bad objects during the choice task for each scene and six pairwise *t* tests for comparison across scenes. Therefore, statistical significance for the post hoc test was set at *α* = 0.05/12 with Bonferroni’s correction.

A sliding time window approach was employed to determine when the neuronal responses to good and bad objects began to diverge significantly in Clusters 1–3 during the choice task. A window size of 50 ms was used, and the window was advanced in 1 ms increments from 575 ms before to 575 ms after the object onset. For each window, the normalized mean neuronal activity toward good and bad objects of individual neurons was calculated, and a paired-sample *t* test was performed between the two conditions. The significance level was set at *α* = 0.05. A significant divergence in neuronal activity was defined as occurring when the *p* value remained below 0.05 for at least 10 consecutive windows. This criterion was applied to reduce the likelihood of false-positive errors. Finally, the time points of significant differences in neural activity were visualized as magenta-colored markers in the panel for each condition, with the first time points that reached significance for 10 consecutive windows shown as vertical dotted lines (Fig. 5*C*,*F*,*J*).

Figure 6, *B*, *E*, and *H*, shows the comparison of neuronal activity at saccade onset for Scene 1 during the choice task. Given that comparisons of the mean neuronal activity during the postobject onset period showed no significant differences for the four scene conditions, analysis of the perisaccade onset period was only performed for the Scene 1 condition. We employed a GLMM to investigate differences in neuronal activity during saccade onset. The full and null models used for the comparison are as follows:

$$\begin{array}{rl} & \mathrm{Full}\;\mathrm{Model}:\;\mathrm{Neuronal}\;\mathrm{Activity}∼\mathrm{Value}\times \mathrm{Direction}\\ & \;+(1|\mathrm{monkey}\_\mathrm{ID})+(1|\mathrm{monkey}\_\mathrm{ID}:\mathrm{Neuron}\_\mathrm{ID}),\\ & \mathrm{Null}\;\mathrm{Model}:\;\mathrm{Neuronal}\;\mathrm{Activity}\\ & \;∼(1|\mathrm{monkey}\_\mathrm{ID})+(1|\mathrm{monkey}\_\mathrm{ID}:\mathrm{Neuron}\_\mathrm{ID}), \end{array}$$

where NeuronalActivity refers to the mean PSTH of individual neurons during 200 ms beginning 100 ms after saccade onset in Scene 1; Value (good vs bad) and Direction (contralateral vs ipsilateral) are the fixed effects; and monkey_ID and Neuron_ID are the random effects. The level of statistical significance for the model comparison was set at *α* = 0.05. We performed six pairwise *t* tests for comparison of mean neuronal activity for good and bad objects during the choice task, and statistical significance for the post hoc test was set at *α* = 0.05/6 with Bonferroni’s correction.

For further analysis, a series of neuronal data analyses were conducted to ascertain the involvement of the recorded neuronal activity in facilitating saccades (Fig. 7). Initially, the data recorded from each neuron were segmented into four groups based on the saccade reaction times in each trial under the four conditions (contralateral/ipsilateral vs good/bad object). These groups were arranged in ascending order of saccade latency, with Group 1 having the shortest latency and the subsequent groups having progressively longer latencies. The average neuronal activity of each group was calculated. This calculation had a 200 ms window, from 100 to 300 ms following object presentation, and the resulting average neuronal activity was standardized using a *Z*-transformation. The correlation coefficients for individual neurons was computed to explore the relationship between saccade latency and neuronal activity. The coefficients were designed to assess the correlation between the group order (from Groups 1 to 4) and mean neuronal activity. Finally, Wilcoxon signed-rank tests were used to determine whether the median of the correlation coefficients differed significantly from zero across the datasets to establish the statistical significance of our findings.

Figure 8, *E*, *H*, *K*, *N*, *Q*, and *T*, shows the comparison of neuronal activities at target onset while monkeys rejected bad objects by suppressing the saccade to a presented bad object (stay) or returning the gaze to the original center point without fixation for >400 ms after the saccade to the target (return) during the choice task and at object onset during the fixation task. By comparing the neural activities for these two cases, we determined whether the observed neural activity was related to the inhibition of the saccade or the rejection of the bad object. It was also determined whether the observed neural activity was related to proactive or reactive inhibition by comparing it with the neural activity during the fixation task. During the fixation task, the same objects were used (their values were constant during Scene 1 of the choice task). We compared the average neural activity for Scene 1 during the choice task when subjects rejected bad objects by return and stay and the neural activity when the objects were presented during the fixation task using GLMM. A GLMM was conducted to test the differences in neuronal activity for the target or saccade onset. The full and null models for the comparison are as follows:

$$\begin{array}{rl} & \mathrm{Full}\;\mathrm{Model}:\;\mathrm{Neuronal}\;\mathrm{Activity}\;∼\;\mathrm{Scene}\times \mathrm{Value}\times \mathrm{Direction}\\ & \;+(1|\mathrm{monkey}\_\mathrm{ID})+(1|\mathrm{monkey}\_\mathrm{ID}:\mathrm{Neuron}\_\mathrm{ID}),\\ & \mathrm{Null}\;\mathrm{Model}:\;\mathrm{Neuronal}\;\mathrm{Activity}\\ & \;∼\;(1|\mathrm{monkey}\_\mathrm{ID})+(1|\mathrm{monkey}\_\mathrm{ID}:\mathrm{Neuron}\_\mathrm{ID}), \end{array}$$

where NeuronalActivity is the mean PSTH of individual neurons during a 200 ms period, beginning 100 ms after target onset; Scene (1–4), Value (good vs bad), and Direction (contralateral vs ipsilateral) are the fixed effects; and monkey_ID and Neuron_ID are the random effects.

The statistical significance for the model comparison was set at *α* = 0.05. Considering that six pairwise *t* tests were performed to compare the normalized neuronal activities for the good and bad objects during the choice task, statistical significance for the post hoc test was set at *α* = 0.05/6 with Bonferroni’s correction.

## Results

### Behavior results in the choice task

During the choice task, the two monkeys demonstrated their ability to differentiate good from bad objects, as shown in Figure 2*A*,*B*. Raincloud plots in Figure 2*A* show the reaction times of both monkeys for the good (saccade for accept) or bad objects (return go saccade for reject) in all four scenes. The cloud portion illustrates the distribution, and the raindrops represent individual data points. The median values and confidence intervals are highlighted in the boxplots (Allen et al., 2021). Table 1 summarizes the number of samples, mean, standard deviation (SD), and 95% confidence intervals (CI) of the reaction times toward good or bad objects in each scene in the neuronal recording sessions.

**Table 1.** Saccade reaction times in each condition of each monkey

	*N*	Mean RT (ms)	SD	95% CI
Monkey C				
Good obj				
ObjA in Scene 1	1,428	160.9	21.6	[159.7–162.0]
ObjC in Scene 2	1,443	162.6	22.4	[161.5–163.8]
ObjE in Scene 3	1,452	169.4	22.1	[168.3–170.5]
ObjF in Scene 4	1,419	165.5	21.4	[164.4–166.7]
Bad obj				
ObjB in Scene 1	1,076	234.3	46.7	[231.5–237.1]
ObjD in Scene 2	1,076	240.0	46.0	[237.2–242.7]
ObjF in Scene 3	1,247	215.0	50.3	[212.2–217.8]
ObjE in Scene 4	1,205	221.3	52.3	[218.8–225.1]
Monkey S				
Good obj				
ObjA in Scene 1	1,466	174.6	20.8	[173.5–175.6]
ObjC in Scene 2	1,482	179.1	22.0	[178.0–180.2]
ObjE in Scene 3	1,490	184.5	21.4	[183.4–185.6]
ObjF in Scene 4	1,476	186.9	24.3	[185.7–188.2]
Bad obj				
ObjB in Scene 1	1,194	302.1	42.5	[299.7–304.6]
ObjD in Scene 2	1,054	311.9	41.2	[309.4–314.4]
ObjF in Scene 3	1,174	291.2	51.8	[288.2–294.2]
ObjE in Scene 4	1,120	299.9	47.4	[297.1–302.6]

SD, standard deviation; CI, confidence interval; RT, reaction time.

Figure 2*B* quantifies the choices made by the monkeys when presented with bad objects. Table 2 lists the number of actions directed toward the bad objects in each scene. Monkeys consistently accepted good objects and frequently made a return (Monkey C: 75.3, 78.1, 91.8, and 85.1% in Scenes 1, 2, 3, and 4, respectively; Monkey S: 74.1, 70.7, 79.7, and 78.2% in Scenes 1, 2, 3, and 4, respectively) and stay (Monkey C: 21.3, 20.2, 6.7, and 11.3% in Scenes 1, 2, 3, and 4, respectively; Monkey S: 23.5, 28.3, 19.5, and 20.1% in Scenes 1, 2, 3, and 4, respectively) for bad objects. The target range was defined as 8° per side square during the task. When the monkeys made a saccade toward the target, their eye position moved out the range for <400 ms, the target disappeared, and the fixation point appeared again.

**Table 2.** Counts of chosen actions for bad objects

	Total	Accept	Return	Stay	Others	Fxbreak
Monkey C						
Scene 1	1,479	0	1,113	314	28	24
Scene 2	1,409	1	1,100	284	15	9
Scene 3	1,396	6	1,282	94	9	5
Scene 4	1,473	6	1,253	167	13	34
Nonswitch (Scenes 1, 2)	2,888	1	2,213	598	43	33
Switch (Scenes 3, 4)	2,869	12	2,535	261	22	39
Monkey S						
Scene 1	1,602	3	1,188	376	30	5
Scene 2	1,504	2	1,063	426	6	7
Scene 3	1,475	1	1,176	287	7	4
Scene 4	1,446	5	1,130	291	15	5
Nonswitch (Scenes 1, 2)	3,106	5	2,351	802	36	12
Switch (Scenes 3, 4)	2,921	6	2,306	578	22	9

Particularly, the monkeys had to wait for 400 ms for the next target to be presented if they rejected bad objects by stay, whereas the waiting time for the next target was shorter than 400 ms if they rejected bad objects by return. This may explain the frequent rejection of bad objects by return. The proportions of stay among the four scenes were significantly higher for Scenes 1 and 2 than for Scenes 3 and 4 for both monkeys (Fisher's test, Monkey C; *p* < 5.55 × 10^−36^, *ϕ* = 0.17, 95% CI: 0.32–0.45; Monkey S: *p* < 1.49 × 10^−8^, *ϕ* = 0.07, 95% CI: 0.62–0.80). This may be attributed to the consistent object values for Scenes 1 and 2 and the alternating values of the objects for Scenes 3 and 4.

### Neuronal activity of striatal neurons during the choice task

To investigate the involvement of striatal neurons in object evaluation, neuronal activity was recorded from the caudate nucleus and putamen of monkeys undertaking the choice task. Through *k*-means clustering based on the responses to contralateral good and bad objects, we quantitatively determined the optimal *K* value by simulating silhouette analysis 5,000 times. A one-way ANOVA followed by post hoc testing revealed that the mean silhouette value at *K* = 3 [0.58 ± 0.0 (SD)] was the highest, leading us to conclude that *K* = 3 was the optimal choice (Fig. 3*A*,*B*). Table 3 details the number of neurons in Clusters 1 (*n* = 38), 2 (*n* = 47), and 3 (*n* = 53). Several task-related neurons were found in the anterior part of the striatum near the anterior commissure for both the caudate nucleus and putamen (Fig. 3*C*).

**Table 3.** Numbers of recorded task-related neurons in the striatum of each monkey

	Cluster 1		Cluster 2		Cluster 3	
	Cd	Put	Cd	Put	Cd	Put
Choice task						
Monkey C	4	14	11	12	9	21
Monkey S	14	6	12	12	14	9
Total	18	20	23	24	23	30
Fixation task						
Monkey C	1	11	8	7	5	17
Monkey S	7	0	1	0	4	0
Total	8	11	9	7	9	17

Cd, caudate nucleus; Put, putamen.

**Figure 4.**
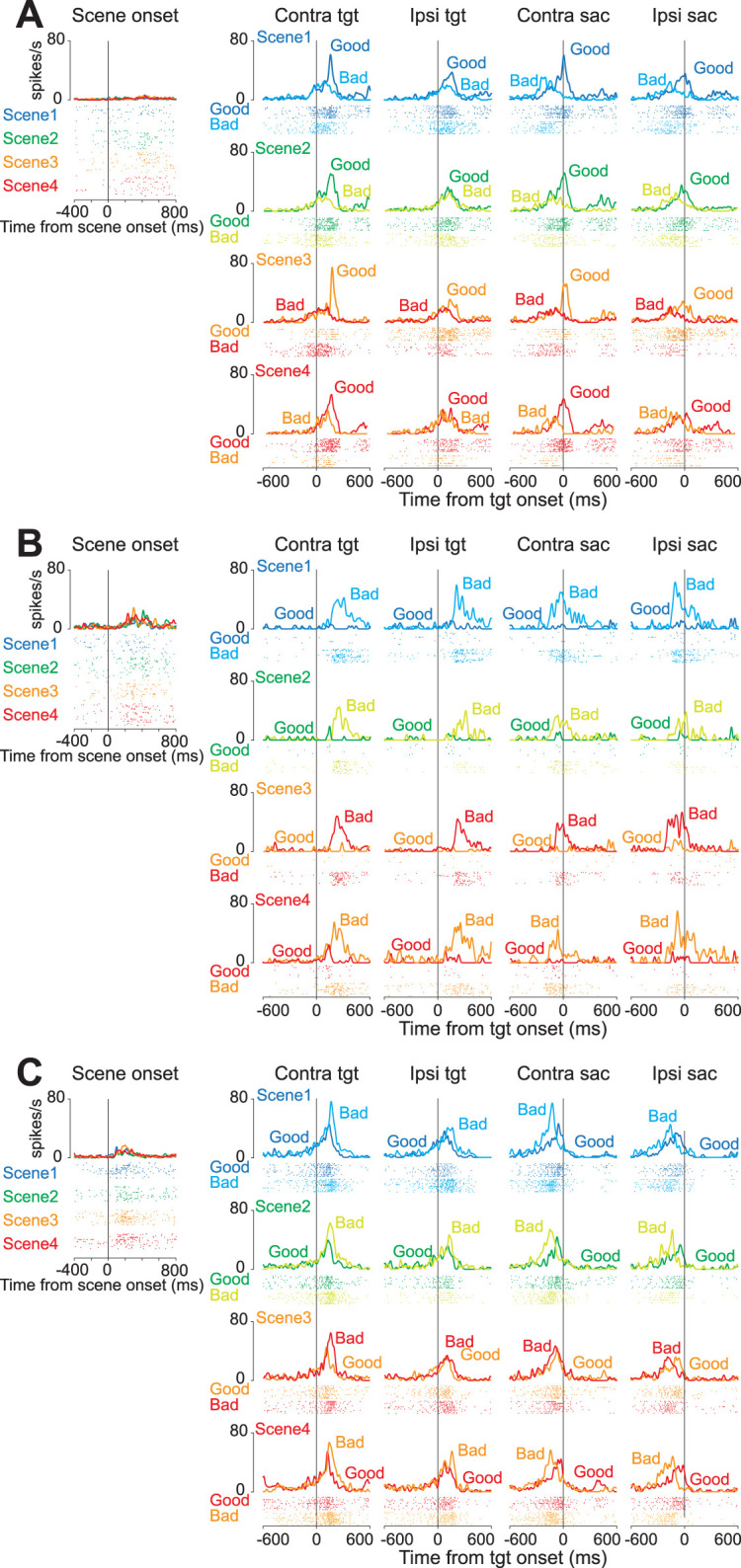
Representative neurons of the three clusters. ***A–C***, The raster plots and histograms of representative neurons of Clusters 1 (***A***), 2 (***B***), and 3 (***C***). Data are aligned with the scene onset, target onset, or saccade initiation (vertical line in each panel). Traces in different colors indicate the spike density for good or bad objects in each scene.

Figure 4*A* shows a representative neuron from Cluster 1. This neuron increased the firing rate more frequently when a good object was presented for all the scenes than when a bad object was. In contrast, one representative neuron in Cluster 2 exclusively increased the firing rate in response to bad objects (Fig. 4*B*). The example neuron of Cluster 3 showed a similar response only after the target onset for the good and bad objects, and the neuronal activity for the bad objects was higher than that for the good objects.

**Figure 5.**
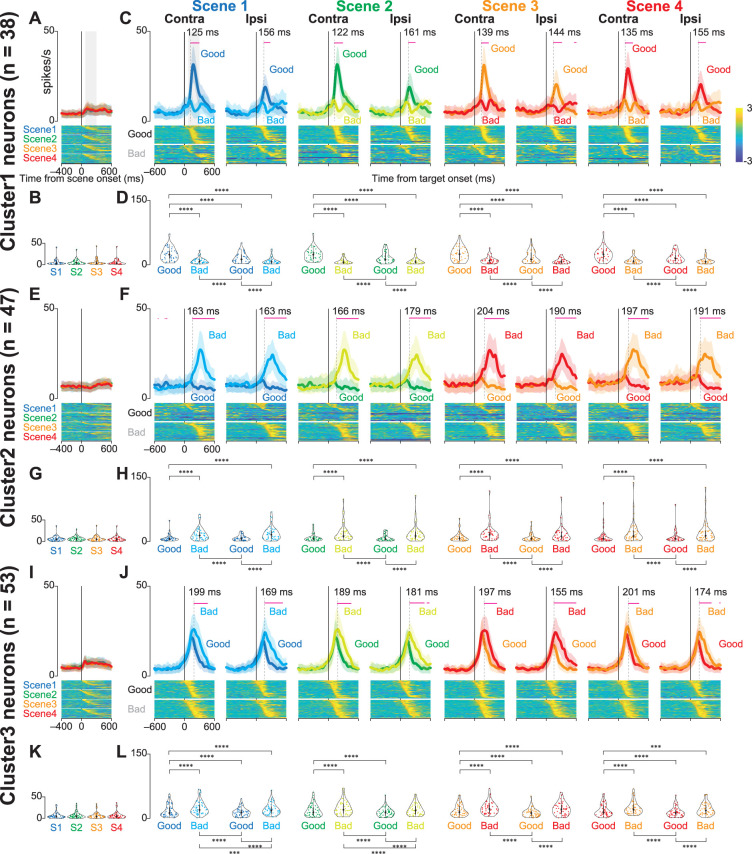
Population activity of three groups of striatal neurons at scene and target onsets during the choice task. ***A***, ***E***, ***I***, Mean population activities aligned at scene onset (Scenes 1–4) during the choice task for the neurons in Clusters 1 (***A***), 2 (***E***), and 3 (***I***). The shaded areas indicate ±SEMs. The bottom panels show the color maps of the normalized firing rates of individual neurons. ***B***, ***G***, ***K***, The violin plots of the mean firing rates of individual neurons in Clusters 1 (***B***), 2 (***G***), and 3 (***K***) for each scene onset during the choice task. Neuronal activity is measured for a 200 ms interval beginning from 100 ms after scene onset (gray rectangle in ***A***). The larger circle indicates the median value, the thick vertical line shows the range between the first and the third quartile, and the thin vertical line indicates the range from the lower to the upper adjacent values. ***C***, ***F***, ***J***, Mean population activities aligned at the contralateral and ipsilateral good and bad target onsets for Scenes 1–4 during the choice task of the neurons in Clusters 1 (***C***), 2 (***F***), and 3 (***J***). The shaded areas indicate ±SEMs (standard error of the mean). The magenta plots indicate the time points when a significant difference between responses to good and bad objects emerged. The vertical dotted lines indicate the first time points that reached significance in 10 consecutive trials. The bottom panels show the color maps of the normalized firing rates of individual neurons. ***D***, ***H***, ***L***, The violin plots of the mean firing rates of individual neurons in Clusters 1 (***B***), 2 (***G***), and 3 (***K***) for each scene onset during the choice task. Neuronal activity is measured for a 200 ms interval beginning from 100 ms after target onset (gray rectangle in the panel of contralateral target onset in Scene 1 in ***C***). The asterisk indicates a significant difference in neuronal activity among the conditions in the choice and fixation tasks (post hoc pairwise *t* tests with Bonferroni’s correction, *p < 0.05, **p < 0.01, ***p < 0.001, ****p < 0.0001). Asterisks are only attached to combinations with significant differences.

Figure 5 shows the population activity of the three clusters at the scene (Fig. 5*A*,*E*,*I*) and target onset (Fig. 5*C*,*F*,*J*). The bottom panels illustrate the normalized neuronal activities of individual neurons in the three clusters at scene onset (Scenes 1–4) and on the presentations of good and bad objects. The violin plots in Figure 5*B*,*D*,*G*,*H*,*K*,*I* show the mean firing rate of individual neurons during the 200 ms from 100 to 300 ms after the scene or target onset. For quantitative analysis, we conducted a parametric bootstrap test for the GLMM and subsequent post hoc pairwise *t* tests with Bonferroni’s correction (see the Materials and Methods section for details). Statistical analysis showed significant differences in neuronal activity when objects were presented during the choice task (post hoc pairwise *t* tests; *p* < 0.0001). The detailed results of the other post hoc pairwise test comparisons are summarized in Tables 4 (Cluster 1), 5 (Cluster 2), and 6 (Cluster 3). There were no significant differences in neuronal activity at the onsets of Scenes 1–4 [parametric bootstrap tests, full model vs null models; *p* = 0.69 (cluster 1), *p* = 0.98 (cluster 2), and *p* = 0.35 (cluster 3)].

**Table 4.** Summary of statistical test to compare the normalized neuronal activity of striatum neurons of Cluster 1 at target onset among conditions during choice task in Figure 5

Cluster 1						
Parametric bootstrap test (*n* = 10,000)	*p*					
Full model vs null model	<0.001					
Post hoc (pairwise *t* test, Bonferroni’s correction)	Mean (SE)	Mean (SE)	*t*	*p*	95% CI	Effect size
Scene 1						
(good, contra) vs (bad, contra)	2.96 (0.13)	1.83 (0.14)	16.61	<0.0001	[0.99 to 1.26]	1.12
(good, contra) vs (good, ipsi)	2.96 (0.13)	2.49 (0.13)	8.69	<0.0001	[0.36 to 0.58]	0.47
(good, contra) vs (bad, ipsi)	2.96 (0.13)	1.80 (0.14)	16.88	<0.0001	[1.02 to 1.29]	1.16
(bad, contra) vs (good, ipsi)	1.83 (0.14)	2.49 (0.13)	−9.03	<0.0001	[−0.80 to −0.51]	−0.65
(bad, contra) vs (bad, ipsi)	1.83 (0.14)	1.80 (0.14)	0.38	=0.71	[−0.13 to 0.20]	0.03
(good, ipsi) vs (bad, ipsi)	2.49 (0.13)	1.80 (0.14)	9.37	<0.0001	[0.54 to 0.83]	0.69
Scene 2						
(good, contra) vs (bad, contra)	2.90 (0.13)	1.71 (0.14)	16.73	<0.0001	[1.05 to 1.33]	1.19
(good, contra) vs (good, ipsi)	2.90 (0.13)	2.56 (0.13)	6.53	<0.0001	[0.24 to 0.45]	0.35
(good, contra) vs (bad, ipsi)	2.90 (0.13)	1.74 (0.14)	16.52	<0.0001	[1.03 to 1.30]	1.16
(bad, contra) vs (good, ipsi)	1.71 (0.14)	2.56 (0.13)	−11.29	<0.0001	[−0.99 to −0.70]	−0.84
(bad, contra) vs (bad, ipsi)	1.71 (0.14)	1.74 (0.14)	−0.31	=0.76	[−0.20 to 0.15]	−0.03
(good, ipsi) vs (bad, ipsi)	2.56 (0.13)	1.74 (0.14)	11.03	<0.0001	[0.67 to 0.96]	0.81
Scene 3						
(good, contra) vs (bad, contra)	2.91 (0.13)	2.02 (0.13)	14.05	<0.0001	[0.77 to 1.02]	0.89
(good, contra) vs (good, ipsi)	2.91 (0.13)	2.59 (0.13)	6.12	<0.0001	[0.22 to 0.43]	0.32
(good, contra) vs (bad, ipsi)	2.91 (0.13)	1.70 (0.14)	16.94	<0.0001	[1.07 to 1.35]	1.21
(bad, contra) vs (good, ipsi)	2.02 (0.13)	2.59 (0.13)	−8.50	<0.0001	[−0.70 to −0.44]	−0.57
(bad, contra) vs (bad, ipsi)	2.02 (0.13)	1.70 (0.14)	3.86	=1.0 × 10^−4^	[0.16 to 0.48]	0.32
(good, ipsi) vs (bad, ipsi)	2.59 (0.13)	1.70 (0.14)	11.91	<0.0001	[0.74 to 1.03]	0.89
Scene 4						
(good, contra) vs (bad, contra)	2.91 (0.13)	1.94 (0.13)	14.82	<0.0001	[0.84 to 1.10]	0.97
(good, contra) vs (good, ipsi)	2.91 (0.13)	2.58 (0.13)	6.10	<0.0001	[0.22 to 0.43]	0.32
(good, contra) vs (bad, ipsi)	2.91 (0.13)	1.81 (0.14)	15.95	<0.0001	[0.96 to 1.23]	1.09
(bad, contra) vs (good, ipsi)	1.94 (0.13)	2.58 (0.13)	−9.40	<0.0001	[−0.78 to −0.51]	−0.65
(bad, contra) vs (bad, ipsi)	1.94 (0.13)	1.81 (0.14)	1.51	=0.13	[−0.04 to 0.28]	0.12
(good, ipsi) vs (bad, ipsi)	2.58 (0.13)	1.81 (0.14)	10.73	<0.0001	[0.63 to 0.91]	0.77

SE, standard error; CI, confidence interval; RT, reaction time.

**Table 5.** Summary of statistical test to compare the normalized neuronal activity of striatum neurons of Cluster 2 at target onset among conditions during choice task in Figure 5

Cluster 2						
Parametric bootstrap test (*n* = 10,000)	*p*					
Full model vs null model	<0.001					
Post hoc (pairwise *t* test, Bonferroni’s correction)	Mean (SE)	Mean (SE)	*t*	*p*	95% CI	Effect size
Scene 1						
(good, contra) vs (bad, contra)	1.58 (0.14)	2.43 (0.13)	−12.82	<0.0001	[−0.98 to −0.72]	−0.71
(good, contra) vs (good, ipsi)	1.58 (0.14)	1.63 (0.14)	−0.66	=0.51	[−0.20 to 0.10]	0.10
(good, contra) vs (bad, ipsi)	1.58 (0.14)	2.50 (0.13)	−13.93	<0.0001	[−1.04 to −0.78]	−0.77
(bad, contra) vs (good, ipsi)	2.43 (0.13)	1.63 (0.14)	12.27	<0.0001	[0.67 to 0.92]	0.93
(bad, contra) vs (bad, ipsi)	2.43 (0.13)	1.80 (0.14)	−1.30	=0.19	[−0.16 to 0.03]	0.03
(good, ipsi) vs (bad, ipsi)	1.63 (0.14)	2.50 (0.13)	−13.40	<0.0001	[−0.99 to −0.73]	−0.73
Scene 2						
(good, contra) vs (bad, contra)	1.75 (0.14)	2.58 (0.13)	−13.59	<0.0001	[−0.96 to −0.72]	−0.71
(good, contra) vs (good, ipsi)	1.75 (0.14)	1.70 (0.14)	0.66	=0.51	[−0.10 to 0.19]	0.19
(good, contra) vs (bad, ipsi)	1.75 (0.14)	2.57 (0.13)	−13.35	<0.0001	[−0.95 to −0.70]	−0.70
(bad, contra) vs (good, ipsi)	2.58 (0.13)	1.70 (0.14)	14.13	<0.0001	[0.76 to 1.00]	1.01
(bad, contra) vs (bad, ipsi)	2.58 (0.13)	2.57 (0.13)	0.20	=0.84	[−0.09 to 0.11]	0.11
(good, ipsi) vs (bad, ipsi)	1.70 (0.14)	2.57 (0.13)	−13.90	<0.0001	[−1.00 to −0.75]	−0.74
Scene 3						
(good, contra) vs (bad, contra)	1.85 (0.14)	2.54 (0.13)	−11.65	<0.0001	[−0.81 to −0.57]	−0.57
(good, contra) vs (good, ipsi)	1.85 (0.14)	1.75 (0.14)	1.48	=0.14	[−0.03 to 0.24]	0.24
(good, contra) vs (bad, ipsi)	1.85 (0.14)	2.45 (0.13)	−9.91	<0.0001	[−0.72 to −0.48]	−0.48
(bad, contra) vs (good, ipsi)	2.54 (0.13)	1.75 (0.14)	12.94	<0.0001	[0.67 to 0.91]	0.92
(bad, contra) vs (bad, ipsi)	2.54 (0.13)	2.45 (0.13)	1.89	=0.06	[0.00 to 0.19]	0.19
(good, ipsi) vs (bad, ipsi)	1.75 (0.14)	2.45 (0.13)	−11.24	<0.0001	[−0.82 to −0.58]	−0.57
Scene 4						
(good, contra) vs (bad, contra)	1.94 (0.14)	2.65 (0.13)	−12.55	<0.0001	[−0.82 to −0.60]	−0.59
(good, contra) vs (good, ipsi)	1.94 (0.14)	1.93 (0.14)	0.20	=0.84	[−0.12 to 0.14]	0.14
(good, contra) vs (bad, ipsi)	1.94 (0.14)	2.64 (0.13)	−12.27	<0.0001	[−0.81 to −0.58]	−0.58
(bad, contra) vs (good, ipsi)	2.65 (0.13)	1.93 (0.14)	12.72	<0.0001	[0.61 to 0.83]	0.84
(bad, contra) vs (bad, ipsi)	2.65 (0.13)	2.64 (0.13)	0.30	=0.76	[−0.08 to 0.10]	0.10
(good, ipsi) vs (bad, ipsi)	1.93 (0.14)	2.64 (0.13)	−12.45	<0.0001	[−0.82 to −0.60]	−0.59

SE, standard error; CI, confidence interval; RT, reaction time.

**Table 6.** Summary of statistical test to compare the normalized neuronal activity of striatum neurons of Cluster 3 at target onset among conditions during choice task in Figure 5

Cluster 3						
Parametric bootstrap test (*n* = 10,000)	*p*					
Full model vs null model	<0.001					
Post hoc (pairwise *t* test, Bonferroni’s correction)	Mean (SE)	Mean (SE)	*t*	*p*	95% CI	Effect size
Scene 1						
(good, contra) vs (bad, contra)	2.66 (0.09)	3.00 (0.08)	−7.86	<0.0001	[−0.43 to −0.26]	−0.35
(good, contra) vs (good, ipsi)	2.66 (0.09)	2.42 (0.07)	4.69	<0.0001	[0.14 to 0.34]	0.24
(good, contra) vs (bad, ipsi)	2.66 (0.09)	2.86 (0.08)	−4.50	<0.0001	[−0.29 to −0.12]	−0.20
(bad, contra) vs (good, ipsi)	3.00 (0.08)	2.42 (0.07)	12.33	<0.0001	[0.49 to 0.68]	0.59
(bad, contra) vs (bad, ipsi)	3.00 (0.08)	2.86 (0.08)	3.41	=7.0 × 10^−4^	[0.06 to 0.22]	0.14
(good, ipsi) vs (bad, ipsi)	2.42 (0.07)	2.86 (0.08)	−9.10	<0.0001	[−0.54 to −0.35]	−0.44
Scene 2						
(good, contra) vs (bad, contra)	2.68 (0.08)	3.00 (0.08)	−7.32	<0.0001	[−0.41 to −0.23]	−0.32
(good, contra) vs (good, ipsi)	2.68 (0.08)	2.44 (0.09)	4.88	<0.0001	[0.15 to 0.34]	0.25
(good, contra) vs (bad, ipsi)	2.68 (0.08)	2.81 (0.08)	−2.67	=7.6 × 10^−3^	[−0.21 to −0.03]	−0.12
(bad, contra) vs (good, ipsi)	3.00 (0.08)	2.44 (0.09)	12.00	<0.0001	[0.47 to 0.66]	0.56
(bad, contra) vs (bad, ipsi)	3.00 (0.08)	2.81 (0.08)	4.69	<0.0001	[0.12 to 0.28]	0.20
(good, ipsi) vs (bad, ipsi)	2.44 (0.09)	2.81 (0.08)	−7.50	<0.0001	[−0.46 to 0.27]	−0.37
Scene 3						
(good, contra) vs (bad, contra)	2.72 (0.08)	2.99 (0.08)	−6.14	<0.0001	[−0.35 to −0.17]	−0.27
(good, contra) vs (good, ipsi)	2.72 (0.08)	2.38 (0.09)	6.82	<0.0001	[0.25 to 0.45]	0.35
(good, contra) vs (bad, ipsi)	2.72 (0.08)	2.88 (0.08)	−4.10	<0.0001	[−0.27 to −0.09]	−0.18
(bad, contra) vs (good, ipsi)	2.99 (0.08)	2.38 (0.09)	12.71	<0.0001	[0.52 to 0.71]	0.61
(bad, contra) vs (bad, ipsi)	2.99 (0.08)	2.88 (0.08)	2.07	=3.9 × 10^−2^	[0.00 to 0.17]	0.09
(good, ipsi) vs (bad, ipsi)	2.38 (0.09)	2.88 (0.08)	−10.77	<0.0001	[−0.62 to −0.43]	−0.53
Scene 4						
(good, contra) vs (bad, contra)	2.73 (0.08)	2.99 (0.08)	−5.95	<0.0001	[−0.34 to −0.17]	−0.26
(good, contra) vs (good, ipsi)	2.73 (0.08)	2.47 (0.09)	5.29	<0.0001	[0.16 to 0.36]	0.26
(good, contra) vs (bad, ipsi)	2.73 (0.08)	2.88 (0.08)	−3.28	=1.1 × 10^−3^	[−0.23 to −0.06]	−0.15
(bad, contra) vs (good, ipsi)	2.99 (0.08)	2.47 (0.09)	11.08	<0.0001	[0.43 to 0.61]	0.52
(bad, contra) vs (bad, ipsi)	2.99 (0.08)	2.88 (0.08)	2.70	=6.9 × 10^−3^	[0.03 to 0.19]	0.11
(good, ipsi) vs (bad, ipsi)	2.47 (0.09)	2.88 (0.08)	−8.49	<0.0001	[−0.50 to −0.31]	−0.41

SE, standard error; CI, confidence interval; RT, reaction time.

Neurons in Cluster 1 were more active when presented with a good object than when presented with a bad object. They were more active when presented with a good object on their contralateral side, suggesting that they were involved in accepting the good object. In contrast, neurons in Cluster 2 were more active when presented with a bad object, suggesting that they were involved in rejecting the bad object. The Cluster 3 neurons showed changes in neural activity immediately after the target was presented, and the temporal changes in neuronal activity immediately after the target was presented were similar for good and bad objects. This suggested that they were involved in the visual response. The changes in neural activity in response to bad objects lasted longer. This may be attributed to the faster saccade reaction times; therefore, the visual response to the good objects was attenuated more quickly.

To estimate the timing when neuronal activity during the choice task significantly differed at the onset of good and bad objects, we performed a time series analysis using 50 ms windows, starting from 575 ms before to 575 ms after object onset. The magenta-colored markers above the traces of population activity indicate the time points that reached statistical significance, while the vertical dotted lines represent the first time points that achieved significance for 10 consecutive windows (Fig. 5*C*,*F*,*J*). This analysis revealed that the time at which neural activity significantly differed between good and bad objects occurred earlier in Cluster 1 than in Cluster 2 under all conditions.

To examine whether the neuronal activity observed during the choice task reflected the visual characteristics or values of the objects presented, we used a set of four scene conditions (Scenes 1–4). There were no significant differences in neuronal activity across scenes. These results suggest that the differences in neural activity observed during target onset are not due to differences in the visual characteristics of the target, but rather differences in the values of the reward for the target.

### Negative correlation between neuronal activity and saccade reaction times

**Figure 6.**
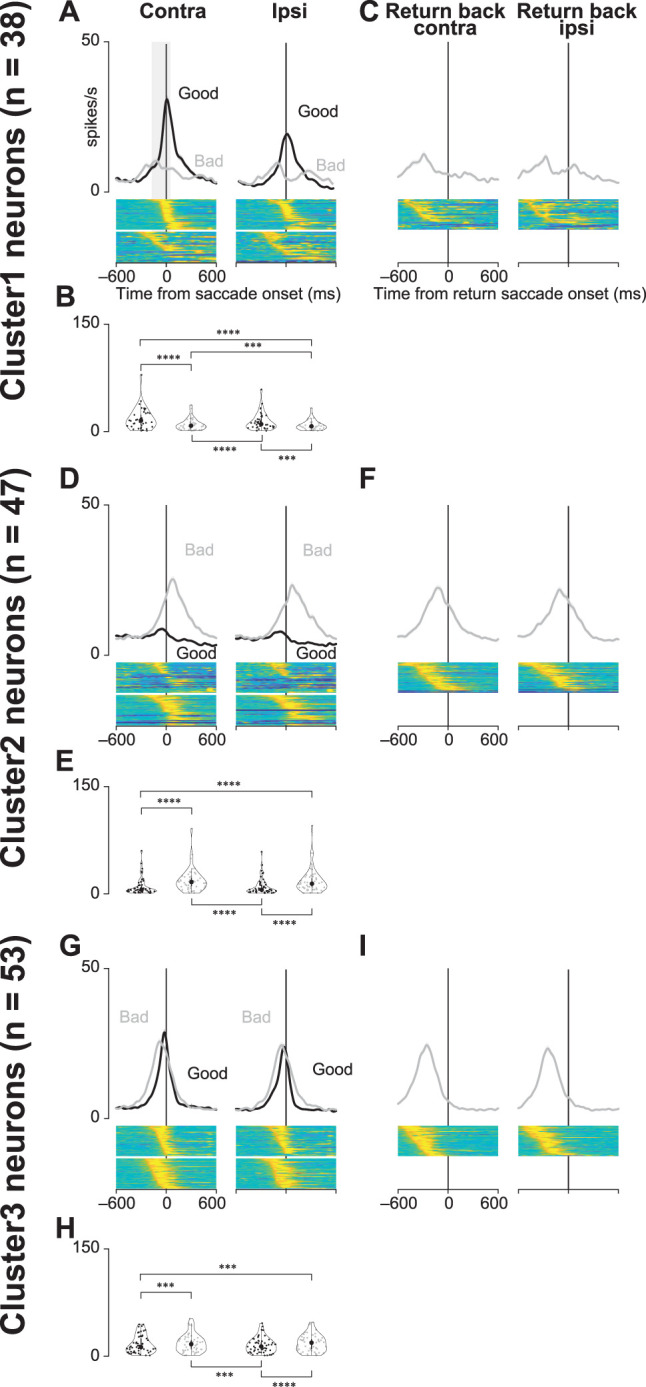
Population activity of three groups of striatal neurons at saccade initiation during the choice task. ***A***, ***D***, ***G***, Mean population activities aligned at saccade initiation toward contralateral or ipsilateral good or bad objects in all scenes during the choice task for the neurons in Clusters 1 (***A***), 2 (***D***), and 3 (***G***). The shaded areas indicate ±SEMs. The bottom panels show the color maps of the normalized firing rates of individual neurons. ***B***, ***E***, ***H***, The violin plots of the mean firing rates of individual neurons in Clusters 1 (***B***), 2 (***E***), and 3 (***G***) when the monkeys make a saccade to the target during the choice task. Neuronal activity is measured for a 200 ms interval from 150 ms before the saccade initiation (gray rectangle in ***A***). The asterisk indicates a significant difference in neuronal activity among the conditions for the choice tasks (post hoc pairwise *t* tests with Bonferroni’s correction, *p < 0.05, **p < 0.01, ***p < 0.001, ****p < 0.0001). Asterisks are attached only to combinations with significant differences. ***C***, ***F***, ***I***, Mean population activities aligned at the initiation of the return back saccade (from the target to the center) after the monkeys make a saccade to bad objects [Clusters 1 (***C***), 2 (***F***), and 3 (***I***)].

Statistical tests were also performed with data aligned at saccade initiation (Fig. 6). There was no obvious difference in the population activity for all three clusters at target onset across the four scene conditions. Therefore, the merged data from Scenes 1, 2, 3, and 4 were used for this analysis. Figure 6, *A*, *D*, and *G*, shows the population activity aligned at the saccade for choosing good targets (accept) or saccades for rejecting bad targets (return go). GLMM and subsequent post hoc pairwise *t* tests revealed significant differences in neuronal activity when the objects were presented during the choice task. Detailed results of other post hoc pairwise test comparisons are summarized in Table 7.

**Table 7.** Summary of statistical test to compare the mean neuronal activity of striatum neurons of Clusters 1, 2, and 3 at saccade onset among conditions during choice task in Figure 6

Cluster 1						
Parametric bootstrap test (*n* = 10,000)	*p*					
Full model vs null model	<0.001					
Post hoc (pairwise *t* test, Bonferroni’s correction)	Mean (SE)	Mean (SE)	*t*	*p*	95% CI	Effect size
(good, contra) vs (bad, contra)	2.38 (0.05)	1.77 (0.07)	7.41	<0.0001	[0.45 to 0.78]	0.61
(good, contra) vs (good, ipsi)	2.38 (0.05)	2.26 (0.06)	1.76	=7.9 × 10^−2^	[−0.01 to 0.27]	0.13
(good, contra) vs (bad, ipsi)	2.38 (0.05)	2.03 (0.06)	4.52	<0.0001	[0.20 to 0.50]	0.35
(bad, contra) vs (good, ipsi)	1.77 (0.07)	2.26 (0.06)	−5.76	<0.0001	[−0.65 to −0.32]	−0.49
(bad, contra) vs (bad, ipsi)	1.77 (0.07)	2.03 (0.06)	−2.99	=2.8 × 10^−3^	[−0.44 to −0.09]	−0.27
(good, ipsi) vs (bad, ipsi)	2.26 (0.06)	2.03 (0.06)	2.86	=4.5 × 10^−3^	[0.07 to 0.38]	0.22
Cluster 2						
Parametric bootstrap test (*n* = 10,000)	*p*					
Full model vs null model	<0.001					
Post hoc (pairwise *t* test, Bonferroni’s correction)	Mean (SE)	Mean (SE)	*t*	*p*	95% CI	Effect size
(good, contra) vs (bad, contra)	1.58 (0.07)	2.85 (0.04)	−17.08	<0.0001	[−1.41 to −1.12]	−1.27
(good, contra) vs (good, ipsi)	1.58 (0.07)	1.47 (0.07)	1.15	=0.25	[−0.08 to 0.30]	0.11
(good, contra) vs (bad, ipsi)	1.58 (0.07)	2.85 (0.04)	−17.06	<0.0001	[−1.41 to −1.12]	−1.27
(bad, contra) vs (good, ipsi)	2.85 (0.04)	1.47 (0.07)	17.77	<0.0001	[1.22 to 1.53]	1.38
(bad, contra) vs (bad, ipsi)	2.85 (0.04)	2.85 (0.04)	0.03	=0.98	[−0.10 to 0.10]	0.00
(good, ipsi) vs (bad, ipsi)	1.47 (0.07)	2.85 (0.04)	−17.75	<0.0001	[−1.53 to −1.22]	−1.38
Cluster 3						
Parametric bootstrap test (*n* = 10,000)	*p*					
Full model vs null model	<0.001					
Post hoc (pairwise *t* test, Bonferroni’s correction)	Mean (SE)	Mean (SE)	*t*	*p*	95% CI	Effect size
(good, contra) vs (bad, contra)	1.57 (0.15)	1.96 (0.15)	−2.95	=3.2 × 10^−3^	[−0.41 to −0.08]	−0.25
(good, contra) vs (good, ipsi)	1.57 (0.15)	1.32 (0.16)	2.68	=7.4 × 10^−3^	[−0.07 to 0.44]	0.25
(good, contra) vs (bad, ipsi)	1.57 (0.15)	1.71 (0.14)	−4.77	<0.0001	[−5.44 to −0.28]	−0.39
(bad, contra) vs (good, ipsi)	1.96 (0.15)	1.32 (0.16)	5.55	<0.0001	[0.32 to 0.67]	0.50
(bad, contra) vs (bad, ipsi)	1.96 (0.15)	1.71 (0.14)	−1.85	=0.06	[−0.29 to 0.01]	−0.14
(good, ipsi) vs (bad, ipsi)	1.32 (0.16)	1.71 (0.14)	−7.29	<0.0001	[−0.81 to −0.47]	−0.64

SE, standard error; CI, confidence interval; RT, reaction time.

Figure 6, *C*, *F*, and *I*, illustrates the population activity aligned at the initiation of return saccades (from the target to the center) when the monkeys rejected bad objects. The modulation of neural activity in Cluster 1 neurons was minimal. The mean neural activities for Cluster 2 and 3 neurons for bad objects started to decrease well before the initiation of the “return back” saccade. This suggests that the average neural activity of Cluster 2 and 3 neurons is more related to the “return go” (initial saccade toward bad objects) or visual response than to the “return back” saccade.

**Figure 7.**
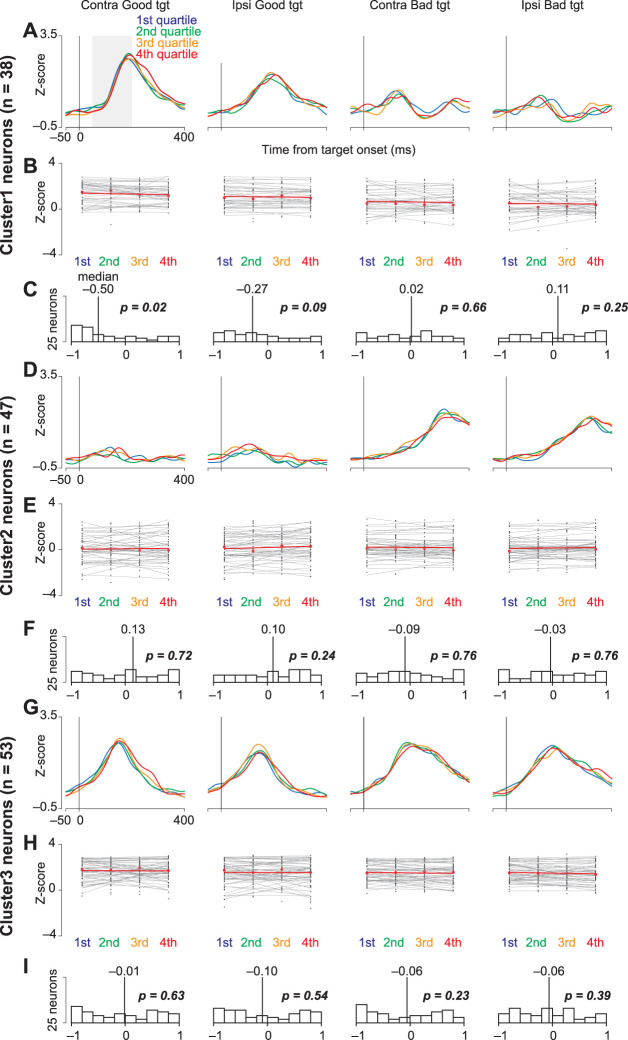
Correlation between neuronal activity and saccade reaction time. ***A***, ***D***, ***G***, Time courses of the population activity of neurons in Clusters 1 (***A***), 2 (***D***), and 3 (***G***) when the monkeys make a saccade toward bilateral good or bad targets. For individual neurons, the data are divided into four groups with an equal number of trials according to the saccade reaction times. The population activity of these four groups is illustrated in the differently colored lines. ***B***, ***E***, ***H***, The regression slopes calculated from the mean neuronal activity classified into four groups according to saccade reaction times for each neuron in Clusters 1 (***B***), 2 (***E***), and 3 (***G***). Neuronal activity is measured for a 150 ms interval from 50 ms after the target onset (gray rectangle in ***A***). ***C***, ***F***, ***I***, Histograms of the distributions of coefficients computed for neuronal activity and individual saccade reaction times [Clusters 1 (***C***), 2 (***F***), and 3 (***I***)]. The vertical lines indicate the median values of each histogram.

This study examined the role of striatal neurons in saccade facilitation. Neuronal data were sorted into quantiles based on saccade latencies and stratified according to the value and direction of object presentation. Figure 7, *A*, *D*, and *G*, shows the collective neural activity for these groups at the target presentation. The correlation between the mean normalized firing rate and saccade latencies for individual neurons is graphically represented in Figure 7*B*,*E*,*H*. Figure 7, *C*, *F*, and *I*, shows histograms of the correlation coefficients for each neuron. Notably, the median correlation coefficient for Cluster 1 neurons, when presented with contralateral good objects, was significantly less than zero (Wilcoxon signed-rank test; *p* = 0.02), suggesting that these neurons influenced the expedited initiation of saccades toward rewarding stimuli presented contralaterally.

### Activated striatal neurons while rejecting the choice are not simply suppressing saccades

**Figure 8.**
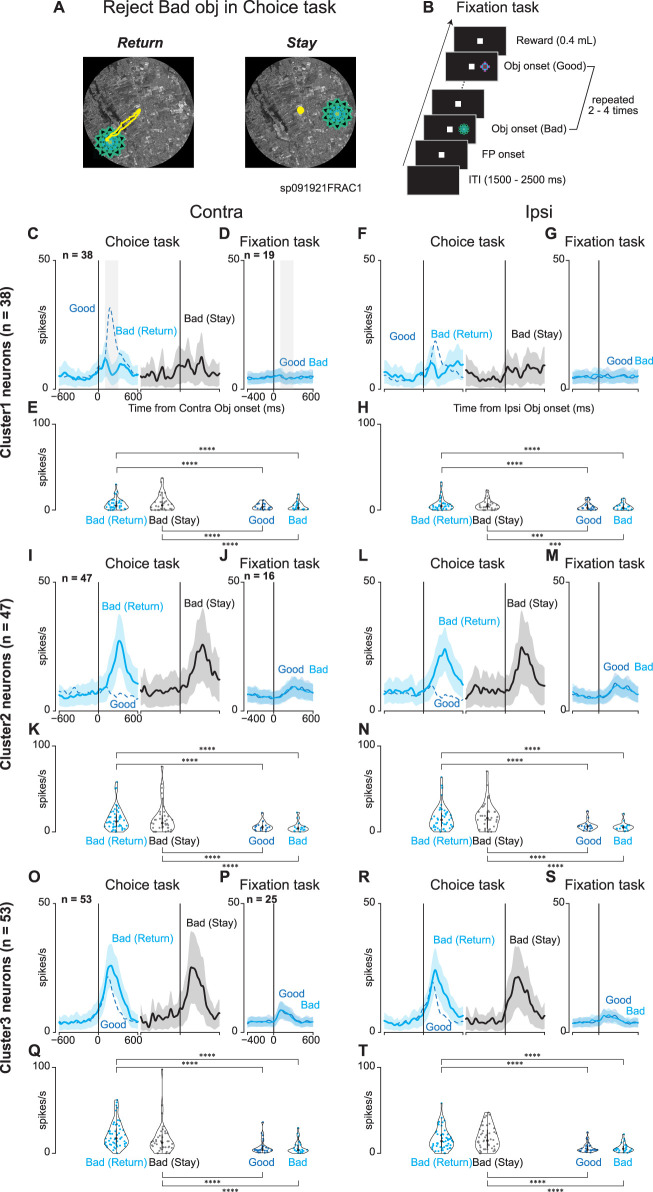
Comparison between return and stay when the monkeys rejected bad objects. ***A***, Examples of eye tracing when a monkey rejects a bad object by making a return saccade (Return) and fixating at the center point (Stay). ***B***, The procedure for the fixation task. During this task, good and bad objects with stable values used in Scene 1 during the choice task are presented on the contralateral or ipsilateral side two to four times in a row. A reward is given at the end if a monkey could keep looking at the center point, suppressing reflexive saccade to the presented object. ***C***, ***F***, ***I***, ***L***, ***O***, ***R***, Mean population activities when the monkeys rejected bad objects in Scene 1 by “return” (cyan-colored line) or “stay” (black-colored line) on the contralateral [Clusters 1 (***C***), 2 (***I***), and 3 (***O***)] or ipsilateral [Clusters 1 (***F***), 2 (***L***), and 3 (***R***)] side. For comparison, the blue dot line represents the mean population activity when the monkeys accept good objects in scene 1. ***D***, ***G***, ***J***, ***M***, ***P***, ***S***, Mean population activities when the monkeys suppressed the reflexive saccade to the presented good objects (blue-colored line) or bad objects (cyan-colored line) at the contralateral [Clusters 1 (***D***), 2 (***J***), and 3 (***P***)] or ipsilateral [Clusters 1 (***G***), 2 (***M***), and 3 (***S***)] side during the fixation task. ***E***, ***H***, ***K***, ***N***, ***Q***, ***T***, The violin plots of the mean firing rates of individual neurons during the choice task [Clusters 1 (***E***), 2 (***K***), and 3 (***Q***)] and the fixation task [Clusters 1 (***H***), 2 (***N***), and 3 (***T***)]. Neuronal activity is measured for a 200 ms interval from 100 ms after object onset (gray rectangle in the panel of contralateral target onset in ***C*** or ***D***). The asterisk indicates a significant difference in neuronal activity among the conditions for the tasks (post hoc pairwise *t* tests with Bonferroni’s correction, *p < 0.05, **p < 0.01, ***p < 0.001, ****p < 0.0001). Asterisks are attached only to combinations with significant differences.

Our analysis further probed activation patterns of the striatal neurons during active rejection of choices, distinguishing between “return” (making a saccade toward but not fixating on the bad object) and “stay” (maintaining gaze near the center point), as shown in Figure 8*A*. This differentiation was crucial as it indicated whether neuronal activity merely reflected saccade suppression or a more complex cognitive process. Utilizing the fixation task (Fig. 8*B*) for neurons that exhibited modulated activity during the choice task, we implemented a parametric bootstrap test for a GLMM and conducted post hoc pairwise *t* tests with Bonferroni’s correction to compare the activities across conditions.

No notable differences were found between the neuronal activities for the “return” and “stay” decisions or the good and bad objects during the fixation task. However, the observed modulation of neurons in Clusters 1–3 during the choice task diminished during the fixation task. This attenuation highlights a potential difference in the roles of neurons. Although their activity decreased during the reactive inhibition required during the fixation task, it was more pronounced during the choice task when proactive inhibition was necessary. These findings, as summarized in Table 8, suggest that neurons in Cluster 2 are particularly active during the inhibition process, as they show similar activity patterns irrespective of “return” or “stay” decisions. The reduced activation during the fixation task implied that these neurons were less involved in simple motor suppression and more involved in complex goal-directed behaviors. This distinction underscores the potential role of striatal neurons in proactive inhibition, wherein they contribute to the selection or rejection of actions to achieve the desired outcome.

**Table 8.** Summary of statistical test to compare the normalized neuronal activity of striatum neurons of Clusters 1, 2, and 3 among Return, Stay during choice task, and fixation task in Figure 8

Cluster 1						
Parametric bootstrap test (*n* = 10,000)	*p*					
Full model vs null model	<0.001					
Post hoc (pairwise *t* test, Bonferroni’s correction)	Mean (SE)	Mean (SE)	*t*	*p*	95% CI	Effect size
Contra						
(Return, choice) vs (Stay, choice)	1.73 (0.15)	1.80 (0.15)	−0.90	=0.37	[−0.24 to 0.09]	−0.08
(Return, choice) vs (Good, fixation)	1.73 (0.15)	1.06 (0.17)	5.46	<0.0001	[0.42 to 0.90]	0.66
(Return, choice) vs (Bad, fixation)	1.73 (0.15)	1.11 (0.17)	5.19	<0.0001	[0.38 to 0.85]	0.62
(Stay, choice) vs (Good, fixation)	1.80 (0.15)	1.06 (0.17)	5.99	<0.0001	[0.50 to 0.98]	0.74
(Stay, choice) vs (Bad, fixation)	1.80 (0.15)	1.11 (0.17)	5.73	<0.0001	[0.46 to 0.93]	0.69
(Good, fixation) vs (Bad, fixation)	1.06 (0.17)	1.11 (0.17)	−0.29	=0.77	[−0.33 to 0.24]	−0.04
Ipsi						
(Return, choice) vs (Stay, choice)	1.69 (0.15)	1.50 (0.15)	2.14	=0.03	[0.02 to 0.38]	0.20
(Return, choice) vs (Good, fixation)	1.69 (0.15)	0.98 (0.18)	5.66	<0.0001	[0.46 to 0.95]	0.71
(Return, choice) vs (Bad, fixation)	1.69 (0.15)	1.00 (0.17)	5.52	<0.0001	[0.44 to 0.93]	0.68
(Stay, choice) vs (Good, fixation)	1.50 (0.15)	0.98 (0.18)	3.89	<0.001	[0.25 to 0.77]	0.51
(Stay, choice) vs (Bad, fixation)	1.50 (0.15)	1.00 (0.17)	3.74	<0.001	[0.23 to 0.74]	0.49
(Good, fixation) vs (Bad, fixation)	0.98 (0.18)	1.00 (0.17)	−0.15	=0.87	[−0.32 to 0.27]	−0.02
Cluster 2						
Parametric bootstrap test (*n* = 10,000)	*p*					
Full model vs null model	<0.001					
Post hoc (pairwise *t* test, Bonferroni’s correction)	Mean (SE)	Mean (SE)	*t*	*p*	95% CI	Effect size
Contra						
(Return, choice) vs (Stay, choice)	2.45 (0.14)	2.49 (0.14)	−0.81	=0.42	[−0.15 to 0.06]	−0.04
(Return, choice) vs (Good, fixation)	2.45 (0.14)	1.50 (0.17)	8.94	<0.0001	[0.75 to 1.16]	0.95
(Return, choice) vs (Bad, fixation)	2.45 (0.14)	1.50 (0.17)	8.94	<0.0001	[0.75 to 1.16]	0.95
(Stay, choice) vs (Good, fixation)	2.49 (0.14)	1.50 (0.17)	9.20	<0.0001	[0.79 to 1.21]	0.99
(Stay, choice) vs (Bad, fixation)	2.49 (0.14)	1.50 (0.17)	9.20	<0.0001	[0.79 to 1.21]	0.99
(Good, fixation) vs (Bad, fixation)	1.50 (0.17)	1.50 (0.17)	0.00	=0.99	[−0.27 to 0.27]	0.00
Ipsi						
(Return, choice) vs (Stay, choice)	2.51 (0.14)	2.52 (0.14)	−0.81	=0.82	[−0.11 to 0.09]	−0.01
(Return, choice) vs (Good, fixation)	2.51 (0.14)	1.59 (0.16)	8.94	<0.0001	[0.73 to 1.13]	0.93
(Return, choice) vs (Bad, fixation)	2.51 (0.14)	1.60 (0.16)	8.94	<0.0001	[0.71 to 1.11]	0.91
(Stay, choice) vs (Good, fixation)	2.52 (0.14)	1.59 (0.16)	9.20	<0.0001	[0.73 to 1.14]	0.94
(Stay, choice) vs (Bad, fixation)	2.52 (0.14)	1.60 (0.16)	9.20	<0.0001	[0.72 to 1.12]	0.92
(Good, fixation) vs (Bad, fixation)	1.59 (0.16)	1.60 (0.16)	0.00	=0.89	[−0.27 to 0.24]	−0.02
Cluster 3						
Parametric bootstrap test (*n* = 10,000)	*p*					
Full model vs null model	<0.001					
Post hoc (pairwise *t* test, Bonferroni’s correction)	Mean (SE)	Mean (SE)	*t*	*p*	95% CI	Effect size
Contra						
(Return, choice) vs (Stay, choice)	2.97 (0.09)	2.93 (0.09)	1.08	=0.28	[−0.04 to 0.14]	0.05
(Return, choice) vs (Good, fixation)	2.97 (0.09)	2.00 (0.11)	12.31	<0.0001	[0.82 to 1.13]	0.98
(Return, choice) vs (Bad, fixation)	2.97 (0.09)	2.02 (0.11)	12.16	<0.0001	[0.80 to 1.11]	0.96
(Stay, choice) vs (Good, fixation)	2.93 (0.09)	2.00 (0.11)	11.38	<0.0001	[0.77 to 1.09]	0.93
(Stay, choice) vs (Bad, fixation)	2.93 (0.09)	2.02 (0.11)	11.22	<0.0001	[0.75 to 1.07]	0.91
(Good, fixation) vs (Bad, fixation)	2.00 (0.11)	2.02 (0.11)	−0.20	=0.84	[−0.22 to 0.18]	−0.02
Ipsi						
(Return, choice) vs (Stay, choice)	2.83 (0.09)	2.76 (0.09)	1.48	=0.14	[−0.02 to 0.16]	0.07
(Return, choice) vs (Good, fixation)	2.83 (0.09)	1.77 (0.12)	12.07	<0.0001	[0.89 to 1.23]	1.06
(Return, choice) vs (Bad, fixation)	2.83 (0.09)	1.78 (0.12)	11.99	<0.0001	[0.88 to 1.22]	1.05
(Stay, choice) vs (Good, fixation)	2.76 (0.09)	1.77 (0.12)	11.15	<0.0001	[0.82 to 1.17]	0.99
(Stay, choice) vs (Bad, fixation)	2.76 (0.09)	1.78 (0.12)	11.06	<0.0001	[0.81 to 1.16]	0.98
(Good, fixation) vs (Bad, fixation)	1.77 (0.12)	1.78 (0.12)	−0.11	=0.91	[−0.24 to 0.21]	−0.01

SE, standard error; CI, confidence interval; RT, reaction time.

**Figure 9.**
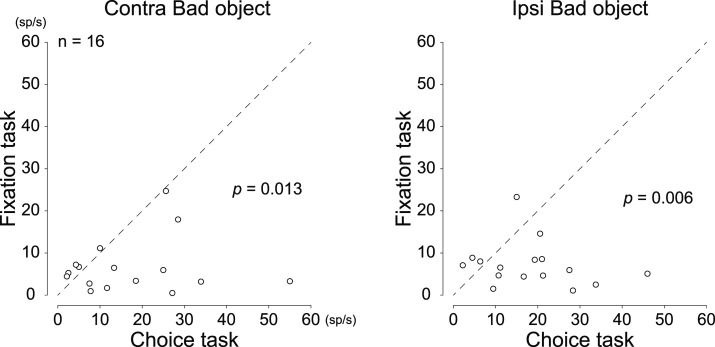
Comparison of the mean firing rates of individual neurons in Cluster 2 during the choice and fixation tasks on bad objects presented. Each plot indicates the mean firing rates of individual neurons during the choice task and fixation task. Neuronal activity was measured for a 200 ms interval from 100 ms after contralateral or ipsilateral bad object onset. The neuronal activity was significantly greater in the choice task when both lateral bad objects were presented (Wilcoxon signed-rank test).

To examine whether neurons in Cluster 2, which were thought to be involved in proactive inhibition due to their activity in the choice task, were also related to reactive inhibition, we compared the mean neuronal activity of individual neurons in Cluster 2 for 200 ms from 100 to 300 ms after bad objects were presented in the choice task to that in the fixation task. Of the 47 neurons in Cluster 2, the neural activity of 16 neurons was recorded in both the choice and fixation tasks, so we compared the mean neuronal activity of these 16 neurons while monkeys performed the two tasks. The overall neuronal activity was significantly greater in the choice task (Fig. 9; Wilcoxon signed-rank test; *p* = 0.013, contralateral bad object; and *p* = 0.006, ipsilateral bad object). This result supports that neurons in Cluster 2 may not be involved in reactive inhibition.

## Discussion

In this study, several neurons within the anterior striatum demonstrated significant activity changed during the choice task, especially when subjects accepted good or rejected bad objects. By clustering the neurons according to their task-related responses, we observed that the activity of one cluster of neurons significantly increased while rejecting bad objects. The rejection strategies of the monkeys, including returning to a central point (return) and maintaining a gaze near it (stay), did not correlate with discernible differences in neural activity. This uniformity suggests that the neuronal processes underlying both rejection strategies are similar with respect to cognitive demand. Furthermore, the subdued neural activity observed during the fixation task relative to the choice task led us to posit that the neuronal activity associated with rejecting bad objects extends beyond mere reflexive saccade inhibition. This appears to be closely related to proactive inhibition, in which monkeys actively dismiss irrelevant options to achieve their goals.

### Involvement of Cluster 2 neurons in cognitive control

Our findings revealed that neurons in Cluster 2 exhibited heightened activity in response to bad objects, suggesting a pivotal role in their rejection. This response pattern aligns with the hypothesis that these neurons are integral to the indirect pathway, which is known to modulate inhibitory control. We observed no significant differences between the neural activities for the “return” and “stay” actions, indicating that these neurons were equally engaged for both rejection strategies.

It is possible that both proactive and reactive inhibition were at work in the choice task in this study. Since the monkeys did not know whether the upcoming object would be good or bad before it was presented, reactive inhibition might have functioned to prevent them from immediately looking at the object. If this were the case, reactive inhibition would likely affect both good and bad objects similarly, leading to similar neural responses to both. However, neurons in Cluster 2 showed little change in neural activity when good objects were presented, suggesting that these neurons were involved in proactive inhibition rather than reactive inhibition.

The time series analysis, which examined when the differences in population activity to good and bad objects became significant, revealed that the initial differences in neural activity were first observed in Cluster 1, followed by significant differences in Cluster 2. This may suggest that the value of the presented object was initially assessed by neurons in Cluster 1, which determined whether to accept the object. If the object's value was judged to be bad, neurons in cluster 2 may have subsequently altered their activity to reject the object.

Furthermore, the subdued neural activity of Cluster 2 neurons during the fixation task underscores their specific involvement in proactive inhibition rather than mere suppression of reflexive saccades. This distinction highlights their role in evaluating and discarding suboptimal choices to facilitate goal-oriented actions, which is a cornerstone of adaptive behavior. These insights into the function of Cluster 2 neurons underscore their contribution to the neural circuitry underlying proactive inhibition and cognitive control. Understanding the dynamics of these neurons sheds light on the mechanisms of cognitive regulation and has potential implications for addressing disorders characterized by impaired decision processes and inhibitory control.

### Anatomical and functional connections of task-related neurons

The task-related neurons identified in this study were predominantly located around the internal capsule of the anterior striatum, specifically in the ventral part of the head and body of the caudate nucleus and the dorsal part of the putamen. These regions receive extensive projections from the frontal eye field (FEF) and the supplementary eye field (SEF), which are critical areas for eye-movement control within the frontal cortex (Huerta et al., 1986; Stanton et al., 1988; Huerta and Kaas, 1990; Shook et al., 1991; Parthasarathy et al., 1992). FEF and SEF neurons are implicated in the cancellation of actions during countermanding tasks, where a planned saccade should be suppressed (Hanes et al., 1998; Stuphorn et al., 2000), as well as for neurons involved in not selecting specific targets in the rostral FEF (Hasegawa et al., 2004). Furthermore, mild microstimulation of the SEF enhances performance during countermanding tasks by delaying saccade initiation (Stuphorn and Schall, 2006).

Given these connections, it is plausible that the neurons in Cluster 2, which are associated with refusal of selection, may be influenced by inputs from these frontal cortex regions. This hypothesis is supported by a recent study that reported intensive modulation of caudate nucleus neuronal activity when monkeys canceled a planned saccade during a stop-signal task, which is analogous to a countermanding task (Ogasawara et al., 2018). These findings suggest that the striatal neurons in Cluster 2 may play a crucial role during the proactive inhibition of actions and contribute to adaptive decisions based on the integration of sensory information and reward-based objectives.

### Role of the striatum during proactive and reactive inhibition

Previous human functional MRI studies have suggested that the striatum is involved in both proactive and reactive inhibition (Vink et al., 2005; Zandbelt and Vink, 2010; Pas et al., 2017). In contrast, the current study identified several neurons in the anterior striatum that were predominantly associated with proactive inhibition, with a notable absence of a response to reactive inhibition. However, this observation does not necessarily imply that the anterior striatum is not involved in reactive inhibition. Our experimental approach initially identified neurons that were responsive to the choice task and subsequently assessed their response to reactive inhibition during the fixation task. We may have overlooked neurons that are specifically responsive to reactive inhibition but not to proactive inhibition. This discrepancy raises the possibility that the striatum may be involved in reactive inhibition but operates through a mechanism different from that of proactive inhibition. Our results suggest a distinct neural basis for these two forms of inhibition within the striatum, suggesting that the cognitive processes underlying proactive inhibition and reflexive response suppression may be mediated by separate neural circuits (Jahanshahi et al., 2015).

### Integration of reactive and proactive inhibition in cognitive control

**Figure 10.**
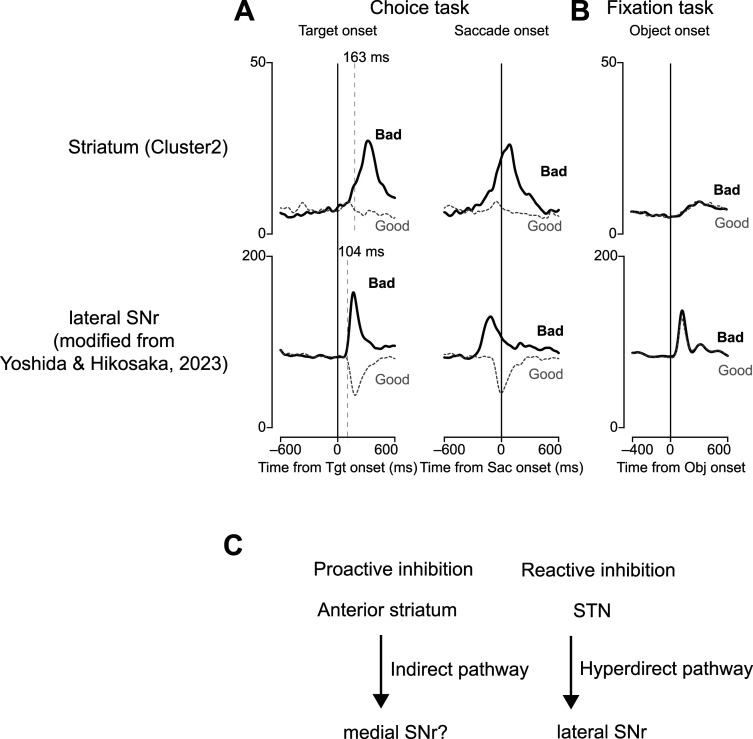
Comparison of putative inhibition-related striatal neurons with lateral SNr neurons. ***A***, Population activity of striatal (Cluster 2) and SNr neurons aligned at the target or saccade onset during the choice task. The vertical dotted lines indicate the first time points that reached significance in 10 consecutive trials. ***B***, Population activity at object onset during the fixation task. ***C***, Hypothetical circuit diagram of two inhibitions.

In our previous research, the neurons in the lateral part of the SNr demonstrated significant alterations in neural activity upon the rejection of bad objects during the choice task and suppression of saccades during the fixation task (Fig. 10*A*,*B*; Yoshida and Hikosaka, 2023). Furthermore, local injections of glutamate receptor inhibitors into the lateral SNr, aimed at inhibiting excitatory projections from the STN, resulted in accelerated saccade responses to bad objects and frequent saccade suppression during the fixation task. These findings suggest the role of the STN→SNr pathway, a component of the hyperdirect pathway, in mediating reactive inhibition.

In the present study, the neural activity of Cluster 2 neurons, which was associated with the rejection of bad objects, peaked near the initiation of the return saccade. On comparing the neuronal activity associated with selective inhibition in the anterior striatum with that in the lateral SNr, we observed a marked difference in temporal dynamics. Specifically, the change in neuronal activity in the anterior striatum was slower than that in the lateral SNr. This distinction suggests that the hyperdirect pathway facilitates the early phase of inhibition, whereas the indirect pathway governs the later phase. This is consistent with the inhibition function hypothesis (Aron, 2011).

This discrepancy in the time course of neuronal activity between these two regions also suggests that the disinhibition of neuronal activity in the striatum is unlikely to directly generate the neuronal activity observed in the SNr. A possible explanation for this is the anatomical projection pattern within these regions. The task-related neurons identified in our study were primarily located in the anterior striatum, which is thought to project to the rostral part of the SNr (Szabo, 1970; Smith and Parent, 1986; Yasuda and Hikosaka, 2015). Based on this anatomical consideration, it is plausible that reactive inhibition involves the lateral SNr, whereas proactive inhibition may be more closely related to the rostral SNr (Fig. 10*C*). This hypothesis is consistent with the distinct temporal profiles of neuronal activity observed in this study. Further investigations are warranted to elucidate the specific neural circuits involved in different types of inhibition.

### Limitation

The current study has a limitation that warrants discussion. While our classification by clustering method and the observed characteristics of neural activity across multiple tasks suggest that one cluster of neurons in the anterior striatum may be involved in proactive inhibition, we did not demonstrate a causal relationship. Ideally, if we could show that inactivating the neuronal activity of Cluster 2 neurons specifically prevents proactive inhibition in monkeys, this would provide definitive proof that the anterior striatum is involved in proactive inhibition. However, achieving this with current technology is challenging. In the future, if the molecular biological characteristics of Cluster 2 neurons beyond their neural activity can be identified, it may become possible to selectively manipulate the neural activity of Cluster 2 neurons using advanced genetic tools, thereby demonstrating a causal relationship.

### Conclusion

We identified a distinct group of neurons in the anterior striatum that demonstrated significant changes in neural activity during the rejection of a bad object as part of a choice task. These neurons demonstrated similar activity patterns when the object was rejected following and without a saccade, suggesting that their role extends beyond the mere suppression of saccadic movements. They are actively involved in the process of choice rejection, irrespective of the method of rejection. Furthermore, the minimal change in neuronal activity observed during the fixation task indicates that these neurons are not primarily engaged during reactive inhibition. Their activities are more consistent with proactive inhibition, which focuses on discarding unnecessary options to achieve a specific goal. These findings clarify the neural substrates of cognitive control, emphasizing the importance of the anterior striatum in orchestrating goal-directed behavior and highlighting the distinct mechanisms underlying different types of inhibition within the brain.
